# Predictors of User Engagement With Facebook Posts Generated by a National Sample of Lesbian, Gay, Bisexual, Transgender, and Queer Community Centers in the United States: Content Analysis

**DOI:** 10.2196/16382

**Published:** 2020-01-28

**Authors:** William C Goedel, Harry Jin, Cassandra Sutten Coats, Adedotun Ogunbajo, Arjee J Restar

**Affiliations:** 1 Department of Epidemiology School of Public Health Brown University Providence, RI United States; 2 Department of Behavioral and Social Sciences School of Public Health Brown University Providence, RI United States

**Keywords:** health communication, social media, sexual and gender minorities, community networks

## Abstract

**Background:**

Lesbian, gay, bisexual, transgender, and queer (LGBTQ) community centers remain important venues for reaching and providing crucial health and social services to LGBTQ individuals in the United States. These organizations commonly use Facebook to reach their target audiences, but little is known about factors associated with user engagement with their social media presence.

**Objective:**

This study aimed to identify factors associated with engagement with Facebook content generated by LGBTQ community centers in the United States.

**Methods:**

Content generated by LGBTQ community centers in 2017 was downloaded using Facebook’s application programming interface. Posts were classified by their content and sentiment. Correlates of user engagement were identified using negative binomial regression.

**Results:**

A total of 32,014 posts from 175 community centers were collected. Posts with photos (incidence rate ratio, [IRR] 1.07; 95% CI 1.06-1.09) and videos (IRR 1.54; 95% CI 1.52-1.56) that contained a direct invitation for engagement (IRR 1.03; 95% CI 1.02-1.04), that expressed a positive sentiment (IRR 1.11; 95% CI 1.10-1.12), and that contained content related to stigma (IRR 1.16; 95% CI 1.14-1.17), mental health (IRR 1.33; 95% CI 1.31-1.35), and politics (IRR 1.28; 95% CI 1.27-1.29) received higher levels of engagement.

**Conclusions:**

The results of this study provide support for the use of Facebook to extend the reach of LGBTQ community centers and highlight multiple factors that can be leveraged to optimize engagement.

## Introduction

Lesbian, gay, bisexual, transgender, and queer (LGBTQ) individuals in the United States experience significant disparities in physical and mental health and in access to health care relative to their cisgender and heterosexual counterparts [1]. Communication plays an important role in eliminating health disparities [2], but health promotion messages are only effective if they reach and resonate with their target audiences. It has been argued that many communication campaigns create messages that employ the surface structure approach to reach their target audience’s culture by matching messages and channels to observable social and behavioral characteristics of their target audience’s members (eg, through the use of familiar people, music, and language) [2]. Effective messaging will also resonate with the historical, social, psychological, and environmental factors that affect the health and well-being of an audience (known as its culture’s “deep structure”) [2]. This entails knowing the kinds of framings (eg, positive vs negative framing [3], gain vs loss framing [4], and 1- vs 2-sided framing [5]), the contents (eg, mental health and sexual health promotion), format (eg, photo and video), frequency or duration, and the context (eg, social or political issues) of the messages that work best to engage individuals with the recommended health behavior [6]. Organizations, particularly those who may not have the capacity for public health programming, may create health communication materials for LGBTQ communities that may not be attuned to their health, information, and communication needs. As such, these organizations may lack an understanding about which channels, contents, and contexts of communication effectively reach their audiences [7], leading to ineffective messaging that does little to improve the health of LGBTQ individuals.

As affirmative and inclusive health services for LGBTQ individuals are lacking in many locations [8], particularly in rural areas [9], LGBTQ community centers remain important venues for reaching and providing crucial health and social services to LGBTQ individuals [10]. LGBTQ community centers are diverse in terms of their mission and structure, and many are independent nonprofit organizations that aim to provide educational, social, and health programming for their clients [10]. Most LGBTQ community centers are physical venues, but regardless of their physical presence, these organizations rely heavily on social media and other digital platforms to reach their clients [10]. Social media represents a common form of digital networking that LGBTQ individuals engage with frequently. In a national probability sample, lesbian, gay, and bisexual adults in the United States were more likely to have a profile on Facebook and use Facebook on a daily basis compared with their heterosexual peers [11]. A significant body of research has shown that LGBTQ individuals use social media sites for many of the same purposes one may access a community center in person, including providing spaces to explore identity, form communities with their peers, access affirming health resources, and engage in political causes [12-15].

The effective use of social media has become a key priority in public health, particularly in reaching populations (eg, LGBTQ communities) that have been overlooked by conventional non-Web-based public health campaigns [16]. Although the use of social media by community centers and community-based organizations (CBOs) is common, the ability of these messages to reach their intended audience and encourage user engagement is varying [17]. Increasing user engagement has become a primary objective of many social media campaigns, but few studies have sought to identify predictors of user engagement [18,19]. A recent study found that social media profiles focused on sexual health promotion that posted often, engaged with individual users, that encouraged interaction and conversation by posing questions, and that shared multimedia content had higher levels of engagement [18]. In general, the reach of social media posts depends on the ability of content to engage and resonate with users. However, because the content produced by LGBTQ community centers may address sensitive, identity-specific topics, the ability of this content to reach its audience could be further constrained by users’ willingness to disclose their identities by publicly engaging with this content [20-22]. As such, we aimed to understand the predictors of user engagement with Facebook content generated by LGBTQ community centers in the United States.

## Methods

Data were collected from Facebook pages administered by 175 LGBTQ community centers in the United States. Facebook pages were purposively selected based on their inclusion in a national directory of LGBTQ community centers maintained by CenterLink, a member-based coalition of LGBTQ community centers formed with the goal of improving the organizational and service delivery capacities of these centers [23].

On the basis of methods used in prior studies [19], data (eg, posts and metrics of engagement with these posts) were downloaded using Facebook’s public application programming interface (API) accessed through the Netvizz application [24]. Posts made in 2017 (January 1 to December 31) were collected and organized by page and post. At the page level, we identified the number of followers for each page. At the post level, we identified the number of likes, reactions, comments, and shares on each post. The study was considered not to be human subjects research and was deemed exempt from institutional review board review. As an extra precaution on behalf of the users who may have interacted with these posts, the names of the Facebook pages included in this study have been omitted. LGBTQ community centers included in the sample were located in 45 of 50 states as well as the District of Columbia.

The content of each post was then analyzed using informatics-based methods [25]. First, using researcher-generated search terms, we identified posts based on 6 topics (with example keywords for each topic in parentheses), related to the missions of LGBTQ community centers, including posts related to stigma experienced by LGBTQ communities eg, stigma, discrimination, and banned), mental health concerns and services (eg, anxious, depressed, counseling, and therapy), education and skill-building (eg, learn, training, and information), youth development (eg, youths, children, and kids), social programming (eg, support, friend, event, and community), and political engagement (eg, vote, election, government, and law). In addition, we identified posts with LGBTQ identity terms (eg, lesbian, transgender, nonbinary, and nonconforming) and posts with explicit invitations for engagement (eg, comment, like, share, visit, click, and take). Posts were able to be identified as belonging to multiple categories.

Each sentence of each post was scored using the Bing Liu sentiment lexicon [26]. This sentiment lexicon is widely used in sentiment analysis and opinion mining and was selected because it provides a freely accessible word database that assigns positive and negative values to keywords. After each word within each sentence of a post was scored, an average sentiment score was assigned to each post indicating whether the post had an overall negative or positive affect. A score of 0 would represent a post with neutral affect, whereas a positive score represents a post with a positive affect, and a negative score represents a post with a negative affect.

Hierarchical negative binomial regression was used to identify post characteristics associated with greater user engagement. In this analysis, the engagement score generated by Facebook was used as an outcome as this is likely an important variable in their algorithm that determines which posts are made visible most frequently. According to the Facebook API, this score is the combined total number of reactions, shares, and comments on each post. Hierarchical negative binomial regression modeling was selected as the statistical approach for this study because the Facebook engagement count data were overdispersed, highly skewed toward 0 and 1, and came from 175 separate Facebook pages—each with a varying number of Facebook “fans” and with differing rates of activity. Incidence rate ratios (IRRs) were calculated based on these analyses.

## Results

During the study period, 32,014 posts were shared by 175 pages. Overall, each page contributed a median of 151 unique posts (IQR 78-264) and had a median of 3347 fans (IQR 1886-5746). These posts received a combined total of 546,492 likes and 32,353 comments and were shared 108,204 times.

Table 1 provides an overview of the posts analyzed. A variety of post types were utilized, with the majority of posts being photos (48.39%, 15,493/32,014) or links (38.26%, 12,250/32,014). Most posts (65.10%, 20,842/32,014) could be classified as containing content related to one of the 7 searched topics (stigma, mental health, education, youth, identity, social, and politics), with identity-related content (42.73%; 13,680/32,014) and content related to social events and socializing (37.01%; 11,850/32,014) being the most common. Example posts from each content area are displayed in Table 2. A total of 1 in 10 posts (11.10%; n=3556/32,014) contained a direct invitation for engagement. Among all posts, the median sentiment score for these posts was 0.2 (IQR 0.1-0.4). Each post received a median of 4 likes (IQR 1-11) and 0 comments (IQR 0-0) and was shared 0 times (IQR 0-2).

**Table 1:** Characteristics of Facebook posts by lesbian, gay, bisexual, transgender, and queer community centers, 2017 (n=32,014).

Post characteristics		Value
**Post type, n (%)**		
	Link	12,250 (38.26)
	Photo	15,493 (48.39)
	Status	2399 (7.49)
	Video	1872 (5.84)
**Post date (day of week), n (%)**		
	Weekday (Monday-Friday)	26,331 (82.24)
	Weekend (Saturday, Sunday)	5683 (17.70)
**Post content type, n (%)**		
	Stigma	700 (2.18)
	Mental health	857 (2.66)
	Education	3450 (10.77)
	Youth	4237 (13.23)
	Social	11,850 (37.01)
	Politics	1445 (4.51)
Mentions an identity term, n (%)		13,680 (42.73)
Direct invitations for engagement, n (%)		3556 (11.10)
Sentiment score, median (IQR)		0.2 (0.1-0.4)
**Post engagement, median (IQR)**		
	Likes	4.0 (1.0-11.0)
	Reactions	5.0 (2.0-14.0)
	Comments	0.0 (0.0-0.0)
	Shares	0.0 (0.0-2.0)
	Engagement score	6.0 (2.0-17.00)

**Table 2:** Examples of Facebook posts by lesbian, gay, bisexual, transgender, and queer community centers classified by content type.

Content type	Example post
Stigma	“The [blinded local legislature] passed [blinded] earlier this week. This discriminatory legislation seeks to give taxpayer-funded agencies a license to discriminate against LGBTQ people under the guise of religion, the dangerous anti-LGBTQ proposal will now move to the [blinded] senate for consideration.” (A link that received 7 likes, 5 comments, and 391 shares)
Mental health	“Meet our mental health team. With their compassion and expertise, this amazing group is the lifeline to many in our community. This team works tirelessly to assure that each person seeking our services gets the support resources and care they need and, when needed, this team goes above and beyond their daily work providing support and guidance for the staff and volunteers at [blinded]. Our mental health programs include couples and family therapy; suicide prevention; support groups for youth, trans men and women. We could not provide this team or our services without the help of our generous donors. Help us maintain our team and services. Help us keep this essential lifeline open. Click on the link below to give today!” (A photo that received 491 likes, 19 comments, and 8 shares)
Education	“Did you see us last night on [blinded]? They visited us to learn more about the expansion of our [blinded] program for trans and gender non-conforming youth that takes place every Tuesday and Thursday!” (A video that received 98 likes, 10 comments, and 44 shares)
Youth	“[Blinded] is a statewide summit for LGBTQ+ youth! We anticipate over 750+ youth will attend. All LGBTQ+ youth ages 14-18 are welcome. Affirming friends are also welcome. Pre-register for shorter lines the day of. This is a summit where all of [blinded]’s youth and their affirming friends ages 14-18 can gather together to build community and foster creativity and ignite their excitement for the future. Affirming parents, counselors, and school administrators are welcome to attend and will have breakout sessions including a Q&A with executive director [blinded].” (A video that received 183 likes, 18 comments, and 71 shares)
Social	“[Blinded] Pride Week is just around the corner and we are excited to announce 2017’s official theme: Connect. In times of uncertainty, building connections is as vital as it is difficult. This Pride Week, we encourage you to keep celebrating being LGBTQ and take time to reflect and plan and connect. Continue following us on social media for event updates. Learn more and register and volunteer.” (A link that received 274 likes, 31 comments, and 100 shares)
Political	“Click ‘Like’ to thank [blinded] City Council for advancing our proposal to ban conversion therapy! The final vote is next Wednesday at 7pm.” (A link that received 368 likes, 2 comments, and 51 shares)

Table 3 presents the unadjusted and adjusted associations of post type, date, content, and sentiment with the Facebook’s user engagement score. Posts containing photos (IRR 1.07; 95% CI 1.06-1.09), links (IRR 1.23; 95% CI 1.21-1.24), and videos (IRR 1.54; 95% CI 1.52-1.56) received higher levels of engagement compared with posts containing status updates only. Posts on weekends also received higher engagement (IRR 1.07; 95% CI 1.06-1.08) compared with posts on weekdays. A total of 6 of 7 content classifications and direct invitations for engagement were associated with increased engagement, with only educational content receiving less engagement (IRR 0.81; 95% CI 0.80-0.81). In addition, positive sentiment was associated with increased engagement (IRR 1.11; 95% CI 1.10-1.12).

**Table 3:** Associations of post characteristics with user engagement with Facebook posts by lesbian, gay, bisexual, transgender, and queer community centers in the United States, 2017.

Post characteristics		Engagement score, IRR^a^ (95% CI)
**Post type**		
	Status	Reference
	Link	1.23 (1.21-1.24)
	Photo	1.07 (1.06-1.09)
	Video	1.54 (1.52-1.56)
**Post date (day of week)**		
	Weekday (Monday through Friday)	Reference
	Weekend (Saturday and Sunday)	1.07 (1.06-1.08)
**Post content type**		
	Stigma	1.16 (1.14-1.17)
	Mental health	1.33 (1.31-1.35)
	Education	0.81 (0.80-0.81)
	Youth	1.13 (1.13-1.14)
	Social	1.03 (1.02-1.04)
	Politics	1.28 (1.27-1.29)
Mentions an identity term		1.10 (1.10-1.11)
Direct invitations for engagement		1.03 (1.02-1.04)
Sentiment score		1.11 (1.10-1.12)

^a^IRR: incidence rate ratio.

In sensitivity analyses (Table 4), we assessed the association of the post characteristics with specific types of user engagement (likes, comments, and shares). Several differences from the main analyses were found. Posts containing links (IRR 0.59; 95% CI 0.56-0.61) and photos (IRR 0.56; 95% CI 0.53-0.58) received fewer comments than posts containing status updates only. Posts with content regarding mental health (IRR 0.86; 95% CI 0.80-0.92), education (IRR 0.86; 95% CI 0.83-0.89), and posts with more positive sentiment (IRR 0.70; 95% CI 0.67-0.74) also received fewer comments. Third, posts containing photos (IRR 0.88; 95% CI 0.86-0.91) were shared fewer times than posts containing status updates only. Posts on weekends (IRR 0.96; 95% CI 0.94-0.97) and posts with more positive sentiment (IRR 0.65; 95% CI 0.63-0.67) were also shared less often. Posts with educational content received fewer likes and comments but were shared more (IRR 1.13; 95% CI 1.11-1.16).

**Table 4:** Associations of post characteristics with specific types of user engagement with Facebook posts (likes, comments, and shares) by lesbian, gay, bisexual, transgender, and queer community centers in the United States, 2017.

Post characteristics		Likes, IRR^a^ (95% CI)	Comments, IRR (95% CI)	Shares, IRR (95% CI)
**Post type**				
	Status	Reference	Reference	Reference
	Link	1.34 (1.32-1.36)	0.59 (0.56-0.61)	1.16 (1.13-1.20)
	Photo	1.31 (1.29-1.33)	0.56 (0.53-0.58)	0.88 (0.86-0.91)
	Video	1.65 (1.62-1.68)	1.30 (1.23-1.37)	1.11 (1.07-1.15)
**Post date (day of week)**				
	Weekday (Monday through Friday)	Reference	Reference	Reference
	Weekend (Saturday and Sunday)	1.10 (1.09-1.11)	1.01 (0.98-1.05)	0.96 (0.94-0.97)
**Post content type**				
	Stigma	1.13 (1.11-1.16)	1.16 (1.09-1.24)	1.26 (1.22-1.30)
	Mental health	1.32 (1.30-1.35)	0.86 (0.80-0.92)	1.73 (1.68-1.79)
	Education	0.73 (0.73-0.74)	0.86 (0.83-0.89)	1.03 (1.01-1.05)
	Youth	1.13 (1.12-1.14)	1.18 (1.14-1.22)	1.13 (1.11-1.16)
	Social	0.97 (0.96-0.98)	1.32 (1.29-1.36)	1.15 (1.13-1.17)
	Politics	1.19 (1.17-1.20)	1.55 (1.48-1.62)	1.41 (1.38-1.44)
Mentions an identity term		1.10 (1.10-1.11)	1.10 (1.07-1.12)	1.11 (1.09-1.12)
Direct invitations for engagement		0.92 (0.91-0.93)	1.25 (1.21-1.30)	1.59 (1.56-1.62)
Sentiment score		1.68 (1.66-1.71)	0.70 (0.67-0.74)	0.65 (0.63-0.67)

^a^IRR: incidence rate ratio.

## Discussion

The study collected post data from 175 Facebook pages associated with LGBTQ community centers across the United States. In total, these pages had approximately 1.1 million fans and shared over 30,000 posts in the span of a year, receiving nearly 700,000 engagements.

We identified a number of factors associated with increased user engagement, and these findings offer practical recommendations as to how LGBTQ community centers can effectively use Facebook to increase the reach of their messages. First, we found that including multimedia content (eg, photos, videos, and links) was associated with higher user engagement compared with text-only status updates. This finding is consistent with a previous study identifying correlates of user engagement with health-related Facebook posts from CBOs serving gay and bisexual men in British Columbia [19] and with the media richness theory [27], which suggests that media that is able to handle multiple information cues simultaneously, facilitates rapid feedback, and establishes a personal focus will be more effective in communicating its message to its audience. Second, in contrast to this previous research in British Columbia [19], we found that direct invitations for engagement (ie, directly asking users to comment, like, or share a post) were associated with increased user engagement.

The content of posts was also significantly associated with user engagement, where posts related to stigma, mental health, and politics received higher levels of engagement. In line with previous research, these topics represent the “deep structure” of the culture of LGBTQ communities and may, therefore, be most salient to the target audience [2]. We note significant temporal variation in the frequency at which key themes were included in posts. For example, a spike in the daily number of stigma-related posts was observed with the announcement of an executive order banning transgender individuals from military service [28], whereas a spike in the daily number of mental health-related posts was observed with the signing of this executive order [28] (Multimedia Appendix 1). Although we are unable to connect these types of events to increases in user engagement with such posts, the presence of such spikes in content production highlights the responsiveness of LGBTQ community centers in their Web presence to events affecting the well-being of their clients.

These findings should be considered in light of their limitations. First, Facebook pages were selected based on their inclusion in a nationwide member-based directory of LGBTQ community centers in the United States and, therefore, these findings may not be generalizable to LGBTQ community centers in the United States who are not part of this directory and LGBTQ community centers outside of the United States. Second, the keyword-based method for classifying the content of posts render the results subject to measurement error, as the selection of key terms may limit the accuracy of content classification. Future research should use more advanced approaches to classify the content of posts, including topic modeling, a statistical technique aimed at discovering latent semantic structures within extensive bodies of text. Third, although we identified correlates of engagement (including specific types of engagement), further research is needed to better understand how each type of engagement promotes the diffusion of health- and nonhealth-related Facebook content within Web-based LGBTQ communities. Fourth, these analyses do not consider user-specific factors that may be associated with engagement (eg, number of Facebook friends who also “like” a page for an LGBTQ community center, whether an individual chooses to disclose their sexual orientation or gender identity in Web-based spaces). Furthermore, research should study post engagement at the individual level to better understand what types of digital content resonate better with their target audiences. Fifth, because the posts were sampled from Facebook, these correlates of user engagement cannot be extended to other social media platforms (eg, Twitter and Instagram) as these platforms have different processes for post content and engagement. Finally, Facebook uses an algorithm to show a user posts that they are likely to interact with. Given the proprietary nature of this algorithm, we are unable to control for how likely a post was to be seen by a given user and note that users are only able to engage with posts that are shown to them. As such, our analyses are restricted to identifying correlates of user engagement conditional on a post being seen by a given set of users.

These results provide support for the use of Facebook by LGBTQ community centers to extend their reach beyond the falls of physical venues and reach their target audiences and highlight multiple factors that can be leveraged to optimize user engagement and enhance the diffusion of information generated by these crucial community institutions. Furthermore, there is potential for public health as a field to engage with these organizations to further build capacity in using evidence-based communication strategies.

## Appendix

Multimedia Appendix 1 Daily time-series of the number of Facebook posts containing content related to stigma and mental health by LGBTQ community centers in the United States.

## Abbreviations

API: application programming interface
CBO: community-based organization
IRR: incidence rate ratio
LGBTQ: lesbian, gay, bisexual, transgender, and queer
