# Supplementary figures and images for: Predictors of User Engagement With Facebook Posts Generated by a National Sample of Lesbian, Gay, Bisexual, Transgender, and Queer Community Centers in the United States: Content Analysis
Source: JMIR Public Health Surveill. 2020 Jan 28;6(1):e16382. doi: 10.2196/16382 (PMC7013651; doi:10.2196/16382)

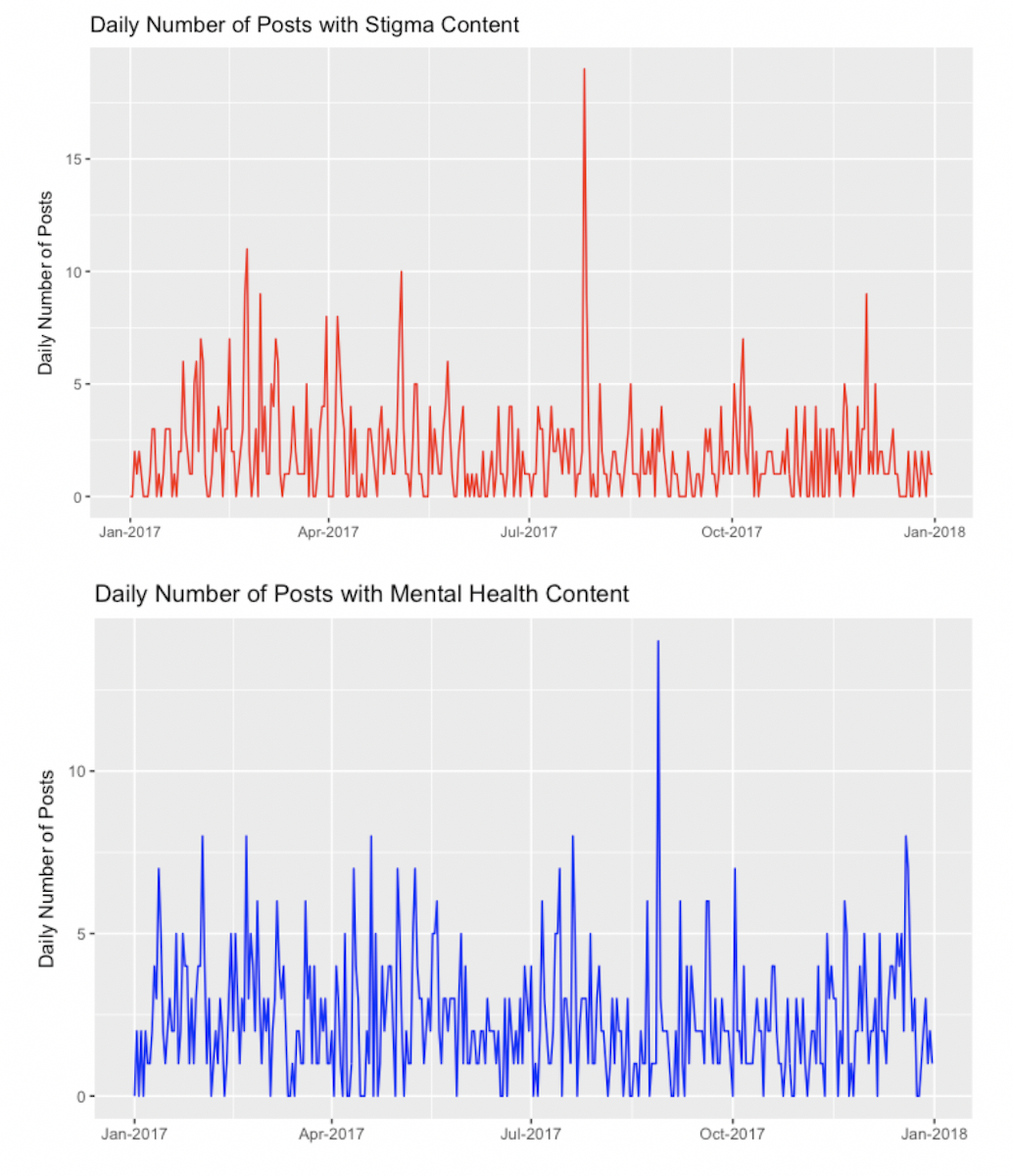

Supplement: Multimedia Appendix 1 [file publichealth_v6i1e16382_app1.png]
